# Intentional or Designed? The Impact of Stance Attribution on Cognitive Processing of Generative AI Service Failures

**DOI:** 10.3390/brainsci14101032

**Published:** 2024-10-17

**Authors:** Dong Lv, Rui Sun, Qiuhua Zhu, Jiajia Zuo, Shukun Qin, Yue Cheng

**Affiliations:** 1 School of Business Administration, Huaqiao University, Quanzhou 362021, China; lvdong@stu.hqu.edu.cn (D.L.); zhuqiuhua6@gmail.com (Q.Z.); zuojiajia@stu.hqu.edu.cn (J.Z.); 23013120015@stu.hqu.edu.cn (S.Q.); 2 Eastern Enterprise Management Research Center, Huaqiao University, Quanzhou 362021, China; 3 College of Architecture and Urban Planning, Tongji University, Shanghai 200092, China; chengyue0798@tongji.edu.cn

**Keywords:** artificial intelligence, stance attribution, service failure, event-related spectral perturbation, mental models

## Abstract

Background: With the rapid expansion of the generative AI market, conducting in-depth research on cognitive conflicts in human–computer interaction is crucial for optimizing user experience and improving the quality of interactions with AI systems. However, existing studies insufficiently explore the role of user cognitive conflicts and the explanation of stance attribution in the design of human–computer interactions. Methods: This research, grounded in mental models theory and employing an improved version of the oddball paradigm, utilizes Event-Related Spectral Perturbations (ERSP) and functional connectivity analysis to reveal how task types and stance attribution explanations in generative AI influence users’ unconscious cognitive processing mechanisms during service failures. Results: The results indicate that under design stance explanations, the ERSP and Phase Locking Value (PLV) in the theta frequency band were significantly lower for emotional task failures than mechanical task failures. In the case of emotional task failures, the ERSP and PLV in the theta frequency band induced by intentional stance explanations were significantly higher than those induced by design stance explanations. Conclusions: This study found that stance attribution explanations profoundly affect users’ mental models of AI, which determine their responses to service failure.

## 1. Introduction

Generative artificial intelligence uses deep learning algorithms to create new text, images, video, or audio content, showing outstanding creative potential and significantly improving user productivity [1]. Generative AI can offer richer and more natural interaction methods for HCI. However, service failures are an unavoidable phenomenon in any high-tech, boundary-pushing domain, especially within the constantly changing and experimenting realm of AI applications [2]. Service failure refers to the gap between users’ expectations of service outcomes and their actual experiences [3]. Service failures are highly likely to lead to a decrease in user trust and may even cause users to abandon the use [4]. The competitive market environment and users’ continuous pursuit of high-standard service experiences make service failure in AI-driven interaction systems a critical issue that could affect brand loyalty and user satisfaction [5]. Unstable user experiences may force consumers to switch to other brands, which is particularly notable in the generative AI market characterized by intense homogeneous competition. Therefore, detailed analysis and improvement in the user experience facing service failures in generative AI are indispensable parts of continuous service quality management and user retention plans.

Research on service failures in the field of human–computer interaction primarily focuses on the discrepancies between system performance and user perceptions. Current studies predominantly concentrate on the reliability of technology and the inconveniences users face due to interruptions [6] as well as investigating the impacts of service failures on user experience from the perspectives of users’ emotional and behavioral responses [7]. However, there is a paucity of research delving into users’ cognitive processing, especially cognitive dissonance indicators in scenarios of service failure. Cognitive conflict may be related to users’ mental models, as a discrepancy between users’ mental models of AI and the actual capabilities of currently generated AI [8] leads to a difference between user expectations and actual services. Moreover, a deeper understanding of the mechanisms of cognitive work is of significant theoretical and practical importance for optimizing human–computer cooperation systems. The cognitive dissonance caused by insufficient services is often latent, as the effectiveness of generative AI technologies typically motivates users to continue their usage [6]. Nonetheless, this subconscious cognitive dissonance and the resulting dissatisfaction can significantly affect their overall experience [9], impacting user loyalty and the brand image of the firm. Particularly in today’s intensely competitive market, exploring the cognitive dissonance experienced by users in the face of service failures with generative AI becomes especially crucial, not only for the continuous iteration and optimization of generative AI products themselves but also for practically improving user experiences and thereby increasing market share.

Generative Artificial Intelligence (AI), fundamentally differs from most historical instances of automation technologies [10], demonstrating a significant improvement in handling complex, emotionally charged tasks. Such tasks, traditionally thought to be challenging for AI technology, have seen many exciting applications in empathy, emotional expression, and the creation of emotion-related content through generative AI [11]. Emotional tasks, inherently different from mechanical tasks, are particularly sensitive in human–computer interaction, encompassing but not limited to social interaction, emotional communication, and the perception of companion robots [12], involving factors such as subjectivity, diversity, uncertainty, and unpredictability. The failure of emotion-oriented services has a different impact on consumer psychology compared to the failure of mechanical tasks. Users may lower their expectations, believing that AI lacks true empathy and emotional understanding capabilities [13]. Therefore, the type of tasks handled by generative artificial intelligence is crucial, as it determines users’ expectations and standards toward the service, as well as the consequences and impacts of service failures. Despite the potential demonstrated by generative artificial intelligence in dealing with emotional tasks, current research on user experience under different tasks remains largely unexplored.

In the field of human–computer interaction, stance attribution refers to how people interpret and predict the behavior of artificial intelligence (AI) or robots [14]. Design stance and intentional stance are shortcuts to understanding and predicting the behavior of complex systems. The design stance focuses more on the system’s function and purpose, whereas the intentional stance assigns to the system a form of “mental” state to predict its behavior [15]. It is about interpreting the behavior of AI or robots either as stemming from the operations of a mind (intentional stance interpretation) or as the result of mechanical design (design stance interpretation) [14]. The intentional stance attributes robot or AI behavior to human-like intentions, desires, or emotions, that is, “mental states” [14]. Such attribution tendencies stem from the theory of mental models, which is the ability of individuals to understand that other intelligent agents have independent minds and, based on this ability, to speculate and comprehend their behavior [16]. Stance attribution involves the process of evaluating and interpreting others’ behavior, and the attitudes and motivations behind it during interactions. When interacting with machines, people attribute certain behaviors to intentions, purposefulness, or emotions, which significantly impact users’ expectations, trust, and ways of interacting with AI [17]. Studies have found that people attribute “stances” to AI and robots, believing these artificial agents possess a degree of autonomous intentions and emotional states, which directly affects the interaction experience with AI [18]. However, existing research has largely focused on users’ attitudes toward stance attribution to AI or robots—how people attribute rational or emotional states to AI. Less explored is how different stance attribution interpretations, from an interaction design perspective, might indeed impact users’ perceptions and experiences.

Furthermore, when performing tasks with a high demand for emotional or mental interpretation, such as mental health counseling or educational tutoring, the use of a “mindful” mechanism by artificial intelligence may have a profound impact on users. Goetz et al. (2003) argued that the design of intelligent systems should match the task [19]. It has been found that the type of task also affects users’ attitudes toward AI [20]. Highly anthropomorphized service robots performing emotionally related tasks (compared to mechanical tasks) can effectively mitigate the negative impact or aversion these robots may cause in consumer reactions [21]. Affective tasks typically involve emotional support and social interaction and are considered to be important for the user’s affective state and social connectedness, while mechanical tasks focus on helping the user to accomplish a specific task, with a predominance of explicitly functional tasks, such as product suggestion or information retrieval [21]. Therefore, it is very necessary to clarify the stance attribution and explanation strategies under different task types to enhance user experience and adjust expectations. Currently, there is a lack of experimental research exploring the joint effect of attribution explanation and task type, with researchers not sufficiently discussing how the two interact.

Previous studies on the failures of robot and artificial intelligence services have predominantly utilized questionnaire self-report methods to collect users’ subjective attitudes and satisfaction levels after service failures [22,23,24]. However, these self-reported data are often based on recollection, rather than immediate responses at the time of the event, and thus may be influenced by recall bias [25]. In the field of generative AI, considering the immediacy of interactions with AI, the instant reactions of user attitudes are very important. Studying the immediate attitudes toward service failures can offer a new perspective for understanding user experiences. Dual-process theory posits that unconscious processes (System 1) are equally important in the attitude formation process and can even be more effective in decision-making and behavior prediction than conscious processing (System 2) [26]. In some complex service interaction scenarios, especially those involving highly personalized and dynamically changing generative AI services, users’ attitudes might be influenced by intuitive reactions related to the specific features of the service, which are processed through System 1. These processes are often rapid, automatic, and difficult to consciously perceive. However, most existing studies have not delved deeply into the micro-psychological processes of users facing AI service failures. It is proposed that approaches based on behavioral and physiological neuroscience, such as motion/eye-tracking, electroencephalography (EEG), and functional near-infrared spectroscopy, should be used in human–computer interaction research to design intelligent systems that can understand and adapt to human users’ needs, emotions, and intentions [27].

To bridge the aforementioned research gap, this study aims to explore users’ unconscious attitudes toward generative AI service failures by employing mental model theory and Dual-Processing Theory. Mental model theory, widely applied in the field of human–computer interaction, reveals how individuals construct models based on experience and knowledge to understand and predict events related to artificial intelligence. It posits that user expectations for interactions are based on their mental models of how they believe the system operates—cognitive frameworks constructed by individuals to predict their surroundings [28]—which subsequently influence user expectations and attitudes toward artificial intelligence [29]. The current research employs an experimental paradigm of event-related potentials with a modified oddball task to detect the unconscious cognitive processes of users when confronted with failures of generative AI services. Event-related potentials are direct responses of the brain to specific events or stimuli, offering a technique for providing real-time data on the cognitive processes of subjects toward complex tasks, as explored by Hinz et al. (2021) in their examination of action planning and outcome monitoring in human–computer interaction [30]. The oddball paradigm, a commonly used ERP experimental design, focuses on studying human attention and cognitive processes. The fundamental concept behind the oddball paradigm is the random insertion of low-probability deviant stimuli into a sequence of high-probability standard stimuli, which can assess abilities such as working memory, stimulus discrimination, and response inhibition [31]. By applying this paradigm in an improved version tailored to human–computer interaction, this approach effectively overcomes the issue of recall biases associated with self-reported studies, revealing the genuine and reliable attitudes of users during the human–computer interaction process.

This study explores whether the design of stance attribution explanations may affect users’ unconscious-level mental models from the perspective of AI psychology, and examines whether task type and stance attribution explanations combine to affect users’ unconscious cognitive conflicts and emotional changes in the event of AI service failures in the light of a new feature of generative AI, i.e., the prominence of affective task capabilities. The innovation of this study lies in discussing stance attribution explanations as a human–machine interaction design that affects users and integrating it with the distinctive feature of generative AI services’ affective capabilities, for the first time combining stance attribution explanations with task types to study users’ unconscious cognitive processes during generative AI service failures. Further innovatively, this study introduces the traditional weird-ball paradigm into the human–computer interaction field, exploring users’ immediate attitudes and micro-level psychological processes toward explanations of generative AI service failures through millisecond-level measurements of brain neurological layers, completely moving away from the recall bias issues brought by traditional self-report research methods. This research offers a new perspective for the application and extension of mental models theory, introduces a new research paradigm to the human–computer interaction field, and provides valuable insights and guidance for designers and developers of generative AI, suggesting the flexible adjustment of stance attribution explanation cues in alignment with task types to minimize users’ cognitive conflicts and emotional dissatisfaction during service failures, thereby enhancing overall user satisfaction and trust, and ultimately increasing market competitiveness.

## 2. Literature Review and Research Hypotheses

### 2.1. Research Related to Failures in Artificial Intelligence Services

As artificial intelligence (AI) penetrates deeper into the service industry, the impact of its failures has attracted widespread attention from researchers. Service failures can affect users’ trust in the service provider, as well as their satisfaction and loyalty [32]. The psychological impacts of AI and robotic service failures are profound and represent an important perspective in the study of human–machine trust dynamics. Current user studies on AI service failures primarily focus on users’ psychological reactions and attitude changes following a failure. It has been found that humorous responses from AI agents can positively influence customers’ tolerance of service failures, with perceived warmth and competence playing mediating roles, and customers’ acceptance and time pressure defining the effectiveness of humor [33]. Sun et al. (2022) discovered that negative technological attributes greatly affect users’ attitudes toward AI assistants, thereby influencing user satisfaction [34]. In scenarios of service failure, consumers are less inclined to share negative word-of-mouth about AI services compared to human services, a phenomenon influenced by the consumer’s perceived connection with the AI and its ability to predict future preferences based on past behaviors [35]. During chatbot service failures, providing customers with the option of human service encourages them to adopt emotion-centered coping strategies, especially among those with a tendency toward low customer engagement and co-production of services, which may lead to an increase in customer aggression [36]. In the context of AI services, the failure of voice AI services significantly promotes customer complaint behaviors, with customer emotion playing a key mediating role in this relationship [37]. Meyer et al. (2022) explored the mechanisms by which poor service performance, corrective strategies, and human–computer interaction designs affect consumer attribution and brand loyalty. The study revealed that when services are successful, consumers tend to view this success as a result of their own decisions; conversely, when service failures are clearly due to robot malfunction, consumers shift the blame to the service provider [3].

In summary, research on artificial intelligence and robot service failures involves both direct interactions and broader societal impacts. As artificial intelligence becomes increasingly complex and integrated across various sectors, understanding and mitigating the risks associated with service failures through design, coping strategies, and ethical considerations will be crucial. However, generative AI, which significantly enhances intelligent and emotional capabilities compared to traditional AI, currently lacks exploration in studies concerning failures in emotive task services.

### 2.2. Research Related to Stance Attribution

Daniel Dennett’s proposed intentional stance and design stance are crucial in human–computer interaction, helping us understand why people view social robots as artifacts yet are willing to interact with them [3]. When users adopt the intentional stance, they interact with artificial intelligence entities under the premise that these AI entities possess beliefs, desires, and consciousness similar to humans. Conversely, the premise of the design stance is the belief that the operation of AI systems is based on algorithms and their creators’ predetermined objectives. Individuals have a tendency to attribute intentional mental states to robotic systems [38]. Scholars have conducted research from a cognitive neuroscience perspective. Bossi et al. (2020) demonstrated through electroencephalogram (EEG) measurements that an individual’s brain activity at rest can predict their tendency to interpret robot behavior as driven by intentionality or mechanical design. This revelation highlights the neural basis of people’s attitudes toward robot behavior, reflecting the deep mechanisms of social understanding within the nervous system [14]. Parenti et al. (2023) revealed that enhanced synchrony in the theta frequency band of the frontal-parietal lobes is associated with an individual’s ability to adopt an intentional stance toward robots. They suggested that long-range theta synchrony could serve as a neural marker of social cognitive processes, a mechanism that can be flexibly applied in interactions between humans and non-human agents such as robots [39]. Roselli et al. (2024) found that individuals with a background in psychotherapy were more inclined than robot experts to attribute intentionality to robots, indicating that people educated in fostering mentalization skills might more readily recognize non-human intelligence in social contexts. This finding was further substantiated by measuring neural activity in a resting state through EEG, revealing an increase in activity in the gamma frequency band (associated with mentalization) among psychotherapists, who are more likely to adopt an intentional stance in interpreting robot behavior. This suggests that educational background and professional training might have a profound impact on how people understand and attribute robot behavior [40].

Effective stance attribution not only enhances user experience but also represents a significant form of endowing AI systems with social properties. However, prior research has predominantly focused on users’ stance attribution attitudes toward AI or robots [41], examining how people attribute rational or emotional states to AI. There has been less exploration of whether different designs of stance attribution interpretations, from an interaction design perspective, truly affect users’ perceptions and experiences.

### 2.3. Research on EEG Correlates in Human–Computer Interaction

Electroencephalography (EEG) technology offers a significant advantage in the field of human–computer interaction by allowing for the measurement of cognitive load. Assessing cognitive load using EEG can help identify complex or unintuitive software functionalities that may lead to cognitive overload in the user experience, thereby supporting the design of more user-friendly interfaces [42]. Research conducted by Perez-Osorio et al. in 2021 demonstrated that, in cooperative scenarios, even robot gazes unrelated to the task can significantly affect people’s inferences about others’ mental states [43]. Specifically, irrelevant gaze signals can disrupt the judgment of mental states by increasing error rates, adding complexity to eye movement paths, and inducing cognitive conflicts in EEG data. Further research has indicated that eye contact with robots not only enhances alpha wave activity in the human brain but also increases the attractiveness of robots, even without altering the effects of gaze cues. This finding underscores the crucial role of eye contact in improving human understanding of non-verbal communication with robots [15]. A study by Parenti et al. in 2023 revealed a connection between the ability to maintain conscious relationships with robots in human–computer interaction environments and increased theta wave activity in the frontal-parietal lobe [39].

Event-Related Spectral Perturbations (ERSP) represent a complex and insightful method used for time–frequency analysis of electroencephalogram (EEG) signals. Observing θ-band ERSPs is indispensable for understanding the brain’s response in tasks that require the processing of conflicting information, such as those involving decision-making, error monitoring, and attentional control [44]. Cognitive conflict arises when an individual encounters incompatible information or demands, necessitating the involvement of control processes to execute coordinated responses. The anterior cingulate cortex (ACC) is considered a critical element for conflict detection and resolution, with θ-band activity serving as a key neural signature of this involvement [45]. Studies have demonstrated that θ-band activity significantly increases when individuals perform tasks that generate conflict, such as the Stroop task or error monitoring paradigms. This reflects the brain’s effort to resolve conflict and maintain cognitive control, managing and overcoming the challenge [46]. Higher theta power is related to the difficulty or effort level of conflict tasks [47]. Changes in theta frequency band initiated by conflict are not only reactive but can also predict subsequent behavioral changes, such as post-error slowing—a phenomenon characterized by increased reaction time following errors [48]. These findings suggest that θ-band activity is closely related to the adaptive regulation of behavior in the face of cognitive conflict. Theta-band functional connectivity is often quantified using phase locking values (PLV), which measure the synchronicity of neural oscillations between different brain regions within the θ band (4–7 Hz). Medial frontal–lateral prefrontal θ-band synchrony can serve as physiological evidence of conflict-induced changes [46].

The integration of electroencephalography (EEG) in human–computer interaction research has demonstrated the interdisciplinary advantages of cognitive science, computer science, engineering, and design. Its high time resolution, effectiveness, portability, and authenticity offer powerful means to deeply explore human–machine interactions and user experience.

### 2.4. Research Hypotheses

**Figure 1:**
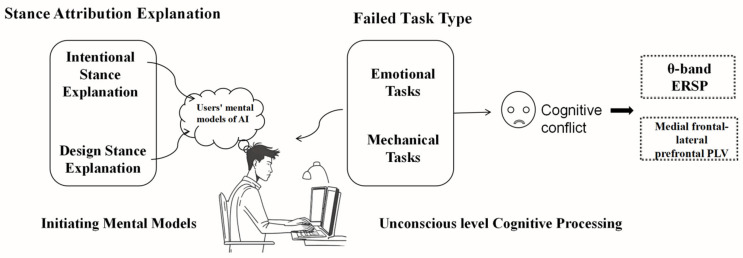
Attribution stance explanations can influence users’ mental models of AI. These mental models lead to different cognitive evaluations by users when various types of generative AI services (emotional or mechanical tasks) fail.

This study explores how the task types of generative AI and the attribution of stances in explanations affect users’ mental models and the resultant cognitive conflict during service failures (see Figure 1). According to research by Pataranutaporn et al. (2023), the activation of beliefs plays a crucial role in the formation of users’ mental models of artificial intelligence. Such mental models are vital for how users understand and respond to AI behaviors [10] and serve as a guide for how they comprehend and interact with AI systems [49]. In human–computer interaction, we hypothesize that through the explanation of stance attribution, beliefs can be implied and guided, thereby adjusting the users’ mental models and encouraging users to understand AI behavior and phenomena naturally—either through the ascription of a “mindful” state (i.e., intentional stance) or from the product design perspective (i.e., design stance). This shift in mental models significantly impacts users’ cognitive evaluations when facing failures in generative AI services. Mental models influence users’ expectations and attitudes toward artificial intelligence [29], leading to different cognitive evaluations of various types of generative AI service failures (affective tasks or mechanical tasks). The content of the AI service, such as emotional communication or expression (affective tasks) and information gathering or processing (mechanical tasks), influences users’ expectations and evaluations of AI systems. Based on the aforementioned theoretical analyses, the following hypotheses are proposed in this study:

**H1:**  The explanation of stance attribution (intentional and design stances) affects users’ unconscious mental models, leading to different cognitive evaluations in various task failure scenarios (affective or mechanical).

**H2:**  The application of an intentional stance explanation significantly enhances users’ tendency to project human psychological states onto AI, especially in emotional services, where service failures lead to more pronounced cognitive conflicts.

**H3:**  The explanation of a design stance emphasizes the functions and design logic of AI, causing users to experience more pronounced cognitive conflicts in the event of mechanical task failures.

## 3. Methodology

### 3.1. Participants

A statistical power of 0.8 and an effect size of 0.25 were set, resulting in a calculated sample size of 24 participants using G*Power. This sample size was also in line with recommendations from previous EEG studies advocating for a minimum of 11 participants [50] to ensure sufficient statistical power. This study, through the participant pool of the Behavioral and Decision-Making Laboratory at Huaqiao University and through social recruitment, enrolled 30 subjects. Four were excluded due to partial electrode damage to the electrode cap, and two participants were excluded due to ERP artifacts exceeding 25% on trial attempts, resulting in a total of 24 valid participants (11 males and 13 females; age 23.791 ± 7.437 years) consisting of college students and individuals from the community. All participants had normal or corrected-normal vision, were right-handed, and had no history of psychiatric disorders. All subjects signed an informed consent form before participating in the experiment and were given a stipend upon completion. This research was approved by the Ethics Committee of Huaqiao University.

### 3.2. Materials

**Figure 2:**
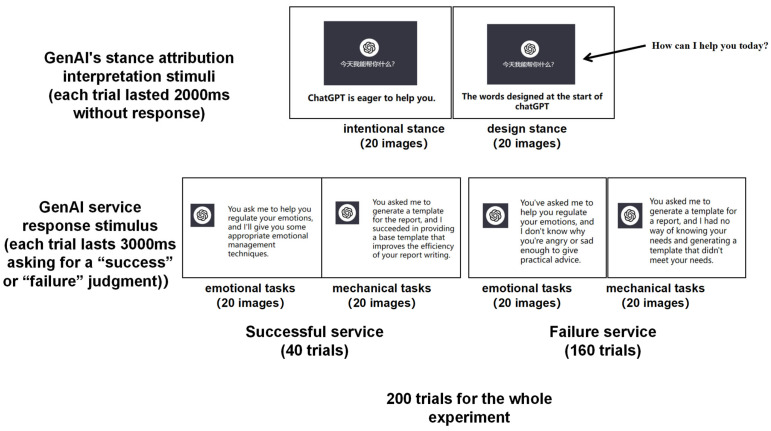
Examples of experimental condition stimuli, examples of specific tasks, and an organizational chart of trial numbers.

The study utilized images from real generative Artificial Intelligence (AI) application scenarios, encompassing platforms including but not limited to ChatGPT3.5 and Bing. The textual explanations accompanying the images were crafted to echo the style of stance attribution scales developed by Marchesi and others in 2019 [51]. Each image was accompanied by two types of explanations: one from a design stance (e.g., “ChatGPT is a designed conversational entity when a problem occurs”) and the other from an intentional stance (e.g., “ChatGPT truly wants to help you, but it made a mistake”). A total of 40 images were organized and reviewed, all of which were audited and certified by four domain experts. The generative AI service responses used in the experiments followed a consistent format, introducing the service (to help users understand the task type) followed by an AI evaluation of the service, i.e., asking users to assess whether the service could successfully complete the task. Service types were divided into emotional tasks and mechanical tasks, with 20 specific task examples for each. The affective and mechanical services were designed with reference to Lin et al.’s (2022) study [21]. Emotional task examples include (“You asked me to design a music playlist to offer comfort and healing”), and mechanical task examples include (“You asked me to generate a report template”). The results of the material screening pre-test by task type *(n* = 264) showed that task types could be effectively differentiated, M = 5.523, SD = 1.653 for affective tasks; M = 2.381, SD = 1.574 for mechanical tasks; t(262) = 15.821, *p* < 0.001. Each service task was designed with both successful and unsuccessful AI responses; hence, there were a total of 40 different AI service response materials. All experimental images and information content were developed by the research team and underwent strict review and approval by four domain experts (see Figure 2).

### 3.3. Procedures

**Figure 3:**
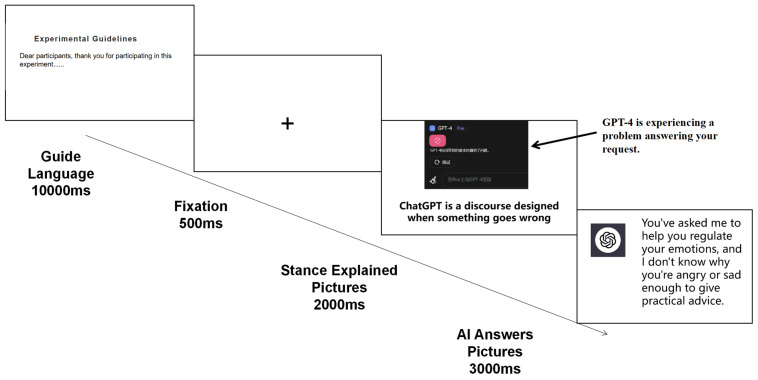
The experimental process included gaze point pictures, AI stance attribution interpretation pictures, generative AI responses to the service, and allowing participants to judge the success of the AI service.

The stimulation process of the experiment was presented using the e-prime program (Psychology Software tools, Pittsburgh, PA, USA), with the entire experimental procedure consisting of 200 trials. The experiment employed a 2 (stance attribution explanation: intentional stance explanation vs. design stance explanation) × 2 (task type: emotional tasks vs. mechanical tasks) within-subjects factorial design using electrophysiological experiment methods. The variant oddball paradigm was the experimental paradigm adopted in this study, whereas the traditional oddball paradigm is based on the prediction of less frequently occurring stimuli, inducing specific brain electrical responses in individuals. This typically involves a high-frequency “standard” stimulus and a low-frequency “target” stimulus, to which participants are required to respond [31]. To overcome the recall bias problem of questionnaires and to better align with human–computer interaction research, this study’s design had participants judge the success or failure of AI services through button presses. Successful (target) stimuli accounted for only 20% of the stimuli, while failed (standard) stimuli made up 80%, with these further divided evenly according to the service task category. This design made the task-type stimuli an unconscious part of the cognitive evaluation for participants, whose main task was simply to differentiate and assess scenarios of service success or failure. In this study, the images answered by AI were marked for subsequent data analysis. Given that the research was conducted in mainland China, we controlled the number of Chinese characters (no more than 10 Chinese words), with the first line containing information about the type of task and the second line containing information about the result of the service. The experiment was carried out in an electromagnetically shielded room treated for sound insulation and reduced light, with participants maintaining a distance of 80 cm from the computer screen. To ensure that each participant was familiar with the experimental tasks, they were given 10 trial runs before the official start. In addition, we verified the participants’ familiarity with the experimental procedure and their reaction time to the experimental materials before the official experiment, confirming that they could effectively receive all the experimental information. The official experiment began with the participants’ consent. The formal experiment was divided into 5 sets, with each set consisting of 40 trials. The experimental flow depicted in Figure 3 starts with each individual trial, where participants first observe a fixation point for 500 ms, followed by a 2000 ms presentation of AI stance attribution explanation images (randomly ordered amongst participants). Finally, participants are presented with and evaluate AI service replies for 3000 ms, needing to judge whether the service was successful (“F” key) or unsuccessful (“J” key).

### 3.4. Data Acquisition and Analysis

This study recorded EEG data using a 64-lead Ag/AgCl electrode cap and Neuroscan Synamp2 Amplifier (Curry7, Compumetrics Neuroscan, Charlotte, NC, USA), with a sampling rate of 1000 Hz. The impedance at all electrode sites was less than 10 kΩ. The FCz electrode served as the online reference electrode, and EEG data were processed through the EEGLAB2023 plugin [52], with re-referencing to the bilateral mastoids, down-sampling to 500 Hz, and filtering in the range of 0.1~40 Hz. Electrical interference at 48~52 Hz was eliminated through band-pass filtering, and artifacts associated with blinking, eye movements, and electromyograms were excluded through independent component analysis.

Theta-band (4–7 Hz) activity, particularly in the frontal midline region (Cz electrode), has long been associated with cognitive control processes, including conflict monitoring and error detection [44]. Increased theta power in this region is thought to reflect enhanced engagement of these cognitive control mechanisms, hence the selection of (C1, Cz, C2, CP1, CPz, CP2) 6 electrodes for the time-frequency analysis in this study. Short Time Fourier Transform (STFT) was performed with a fixed time window of 400 ms, and baseline correction was determined through decibel conversion, using the formula 10×log10 (B(t,f)/A(f)), selecting from −800 to −200 ms for baseline correction. In functional connectivity analysis, data were downsampled to 250 Hz, and peripheral electrodes such as P7, P8, F7, and F8 were excluded to reduce computational load. STFT was applied to the data, with baseline correction from −600 ms to −200 ms. The synchrony in the θ frequency band between the medial and lateral prefrontal cortex can detect unconscious cognitive conflict. The study analyzed the phase locking value (PLV) between FCz (medial frontal electrode) and F6 (lateral frontal electrode) electrode pairs [46].

## 4. Results

### 4.1. Time–Frequency Results

**Figure 4:**
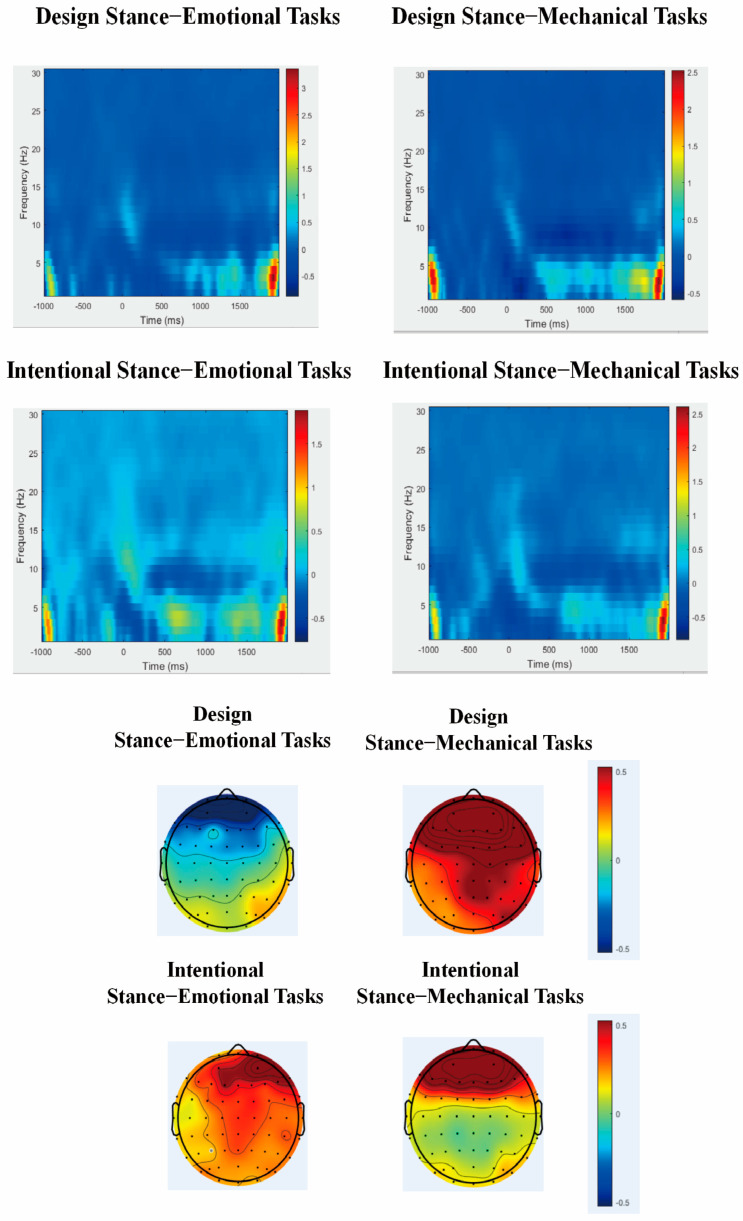
Spectrograms of ERSD induced by cz electrode position in 4 conditions with corresponding topography.

The EEG spectrum was obtained through the Short Time Fourier Transform (STFT), as shown in Figure 4. The time–frequency distribution within the range of 0.1~30 Hz for the Cz electrode is depicted in Figure 4. A 2 (stance interpretation type) × 2 (task type) × 6 (electrode) repeated measures ANOVA on ERSP values in the theta frequency band (4–7 Hz) between 1550 and 1650 ms revealed a significant interaction between task type and stance interpretation type (F(1, 23) = 13.048, *p* = 0.001, ηp2 = 0.362); H1 is supported. No other main effects or interactions were significant.

The simple effects analysis revealed that under the design stance interpretation, the energy induced by failures in emotional tasks (M = −0.089, SE = 0.133) was significantly lower than that in mechanical tasks (M = 0.506, SE = 0.209, *p* = 0.021); H3 is supported. Under the intentional stance, the energy induced by failures in emotional tasks (M = 0.324, SE = 0.151) was marginally significantly higher than that in mechanical task failures (M = 0.020, SE = 0.114, *p* = 0.096); H2 is supported. In the case of failures in emotional tasks, the energy induced by the intentional stance interpretation (M = 0.324, SE = 0.151) was significantly higher than that by the design stance interpretation (M = −0.089, SE = 0.133, *p* = 0.024). In the case of mechanical task failures, the energy induced by the design stance interpretation (M = 0.506, SE = 0.209) was significantly higher than that by the intentional stance interpretation (M = 0.020, SE = 0.114, *p* = 0.025).

### 4.2. Functional Connectivity Analysis Results

**Figure 5:**
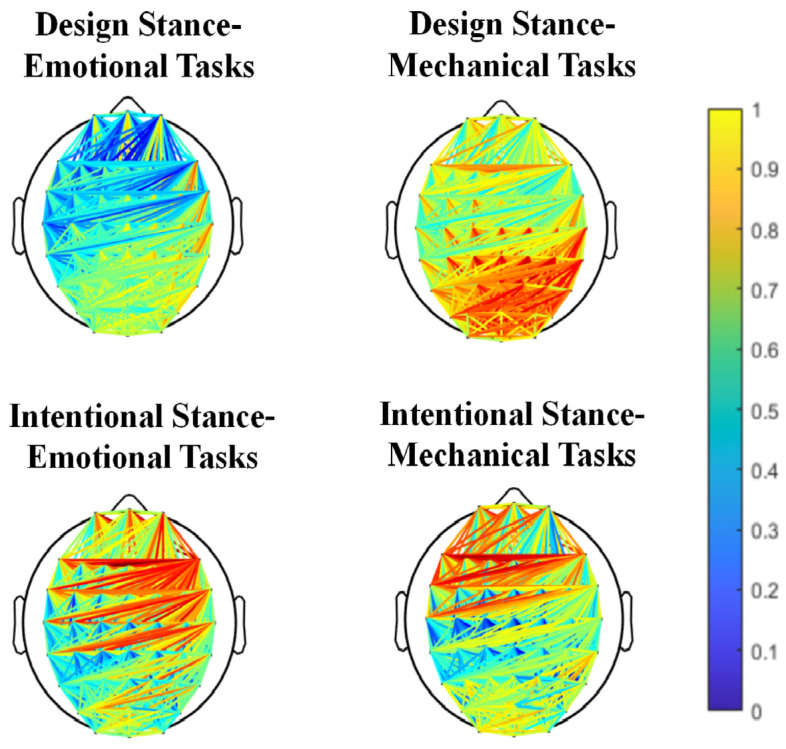
Functional connectivity diagrams in 4 conditions.

To verify the time–frequency results, a functional connectivity analysis was conducted on brain regions related to conflict, as shown in Figure 5. Functional connectivity between FCz and F6 electrodes was evaluated within the 4–7 Hz time–frequency range from 1550 to 1650 ms. A 2 (stance interpretation type) × 2 (task type) × 6 (electrode) repeated measures analysis was performed for the FCz and F6 electrodes in the theta frequency band (4–7 Hz) during 1550–1650 ms. The results for Phase Locking Value (PLV) showed a significant main effect of stance interpretation type (F(1, 23) = 11.546, *p* = 0.002, ηp2 = 0.334) and a significant interaction between task type and stance interpretation type (F(1, 23) = 5.156, *p* = 0.033, ηp2 = 0.183). The main effect of task type was not significant. Pairwise comparisons revealed that the PLV under stance interpretations (M = 0.026, SE = 0.010) was significantly higher than that under design stance (M = −0.013, SE = 0.008, *p* = 0.002). Simple effects analysis showed that under design stance interpretations, PLV values for failed emotional tasks (M = −0.029, SE = 0.011) were significantly lower than those for failed mechanical tasks (M = 0.003, SE = 0.010, *p* = 0.041). Under the intentional stance, there were no significant differences in task failures (*p* = 0.545). For failed emotional tasks, PLV values under intentional stance interpretation (M = 0.031, SE = 0.012) were significantly higher than those under design stance interpretation (M = −0.029, SE = 0.011, *p* < 0.001). There were no significant differences in PLV values between the two types of stance interpretations for failed mechanical tasks (*p* = 0.195).

## 5. Discussion

Few studies have explored the unconscious cognitive conflicts during human–computer interaction processes, nor have they considered the interpretation of stance attribution as a factor influencing human–computer interaction design. To understand people’s reactions when generative AI services do not meet expectations, it is essential to delve into human cognitive processes and our interactions with technology. This research design innovates the oddball paradigm and introduces it to the field of AI psychology, getting rid of the recall bias problem associated with the traditional self-reporting research methodology, and revealing how task-type and stance attribution explanations of generative AI affect users’ attitudes and behaviors when the service fails, as well as the micro-psychological and cognitive processing mechanisms involved in this process.

The theta frequency band (4–7 HZ) ERSP (Event-Related Spectral Perturbation) values represent event-related spectral changes that reflect the brain’s frequency-domain response to stimuli, allowing for analysis of changes in different neural oscillation bands. The theta frequency band (4–7 HZ) is a low-frequency neural oscillation primarily occurring in the frontal midline area, associated with attention, memory, emotional, and other cognitive functions [44]. Increased theta power in this region is thought to reflect increased engagement of these cognitive control mechanisms. Therefore, the observed differences in ERSP values suggest that the interaction between stance interpretation and task type modulates the level of cognitive conflict experienced in response to AI service failures. This study found that the attribution of stance and task type comprehensively affects the induced theta-band ERSP (Event-Related Spectral Perturbation) energy values in users when generative AI services fail. Since the theta-band ERSP is associated with cognitive control and conflict, our findings indicate that under a design stance explanation, cognitive conflict induced by failures in emotional tasks is significantly lower than that in mechanical tasks. However, under an intentional stance explanation, cognitive conflict induced by failures in emotional tasks is significantly higher than that in mechanical tasks. Similarly, in the scenario of failures in emotional tasks, the cognitive conflict induced by failed generative AI services under an intentional stance explanation is far greater than that under a design stance explanation. In instances of mechanical task failures, users experience greater cognitive conflict in response to failed generative AI services explained by a design stance. Our results supported H1, which predicted a significant interaction between task type and stance interpretation type on the ERSP values in the theta frequency band. This significant interaction indicates that cognitive processing of AI service failures is influenced by both stance attribution explanation and task type. This finding aligns with the theory of mental models, which suggests that users’ internal representations of the AI system are shaped by both the task context and the provided explanations, leading to different expectations and responses to system failures. Further, the findings from the simple effects analysis supported Hypotheses 2 and 3. H2 proposed that under the intentional stance interpretation, failures in emotional tasks would induce higher cognitive conflict compared to mechanical tasks, reflected in higher ERSP energy values. This was marginally supported, with energy induced by failures in emotional tasks being marginally significantly higher than in mechanical task failures under the intentional stance. H3 posited that under the design stance interpretation, failures in mechanical tasks would induce higher cognitive conflict compared to emotional tasks. This was supported by the finding that under the design stance interpretation, energy induced by failures in emotional tasks was significantly lower than in mechanical tasks.

Phase Locking Value (PLV) is a metric for quantifying the synchronicity of neural electrical signals, with higher PLV values indicating more effective communication and information integration between brain regions. The synchronization between specific brain areas, such as the Medial Frontal Cortex (MFC) and the Lateral Frontal Cortex (LFC), is considered to be crucial for cognitive functions such as cognitive conflict, error detection, attention, and working memory [46]. Phase Locking Value (PLV) quantifies the synchronization of neural oscillations between brain regions. Increased PLV between the frontal midline (FCz) and lateral prefrontal cortex (F6) in the theta band suggests enhanced communication and integration of information related to conflict monitoring and cognitive control [46]. The observed differences in PLV values therefore indicate that stance interpretation and task type influence the degree of functional connectivity between these regions during the processing of AI service failures. This study employed PLV measurements between the Medial Frontal Cortex (e.g., FCz electrode) and the Lateral Frontal Cortex (e.g., F6 electrode), where the Medial Frontal Cortex is generally associated with error monitoring (e.g., functions of the Anterior Cingulate Cortex, ACC) and conflict monitoring (related to task switching, social signal processing), while the Lateral Frontal Cortex is related to goal setting and maintenance, participating in a broader range of cognitive control. The results of the functional connectivity in this study found that under the interpretation of the design stance, failures in mechanical tasks, as opposed to emotional tasks, elicited neural activity associated with unconscious cognitive conflicts and error monitoring in users. Moreover, the presence of service failures during emotional tasks, as interpreted by the intentional stance compared to the design stance, more profoundly elicited unconscious cognitive conflicts and error monitoring in users. Additionally, these findings confirmed the above-mentioned time–frequency analysis results.

Our research findings suggest that the stance attribution explanations of generative AI during human–machine interaction can significantly influence the mental models and expectations of users toward AI agents, as reflected in neural activity associated with unconscious processes. Stance attribution explanations can be regarded as a guiding mechanism that directs how users perceive and evaluate the actions of artificial intelligence. They profoundly affect the mental models of users’ internal representations, based on which users interpret, predict, and rationalize the behaviors of AI. These mental models play a decisive role in how users react to service failures encountered with AI services. Mental models suggest that individuals construct internal representations of systems based on information and experiences, including artificial intelligence and robotics [3]. Mental models are dynamic, adaptive cognitive structures that help people understand complex systems such as AI agents and interact with them, serving as guides for how they comprehend and engage with AI systems [49]. Mental models can also affect users’ expectations and attitudes toward artificial intelligence [29], thereby influencing cognitive conflicts in users during different task service failures. Research has found that priming statements can influence how individuals construct mental models of generative AI using their past perceptions and expectations [10]. In our study, stance attribution explanations play a similar role, influencing the psychological representations of artificial intelligence among users.

Belief initiation can affect the building of users’ psychological models [10], and within human–computer interactions, stance attribution explanations can perform the function of belief implantation and suggestion, leading users to naturally interpret AI behaviors and phenomena through an “intentional stance”—attributing mental states or from a “design stance” perspective. Task type, referring to the tasks that users perform with AI involving emotional exchange or expression (emotional tasks) or information acquisition or processing (mechanical tasks), influences users’ needs and evaluations of AI [21]. Services oriented toward emotion require empathy, communication, and emotion, while tasks that are tool-oriented are more objective, logical, and quantifiable, not involving emotion [21].

When individuals receive interpretative explanations of AI behaviors in terms of intentions, they tend to project human-like psychological states (such as intentions, desires, and beliefs) onto the AI. During human–computer interactions involving emotional services with AI, if the AI is designed to lean more toward intentional stances, it is easier to evoke mental states associated with interacting with other humans [51]; thus, any failure can trigger more severe cognitive dissonance. Interpretations focusing on the design stance emphasize functional and system design aspects, demonstrating the rationale behind behaviors in a mechanical or predetermined manner [14]. Furthermore, it is commonly believed that robots and mechanical orientation tasks are more appropriately linked [53]. Explanations based on the design stance facilitate people in connecting AI behavior with explicit programming and specific functionalities. Hence, any failure in mechanical tasks can lead to significant cognitive discord among users. Intelligent system designers and developers should delve into users’ micro-psychological understandings to create AI products that better align with user mental models.

User characteristics, such as prior experience with AI technology [54], mental distance [55], technical proficiency [56], and individual differences in attachment styles [57], may significantly influence how users interact with AI systems and react to service failures. Prior research has shown that positive prior experiences with AI technology can play a positive role in human–computer interaction, while perceived risk acts as a deterrent [54]. In addition, personality traits may influence users’ perceptions and interactions with AI agents [58]. Desirability and youthfulness predict a more positive attitude toward AI technology, while sensitivity to conspiracy theory beliefs leads to a more negative attitude [59]. Customer inoculation measures can be effective in increasing satisfaction with service remedies after AI service failure [55]. While our study focuses on the neural mechanisms behind cognitive conflicts when AI services fail, different user characteristics may modulate these neural responses. Combining user experience assessment with AI, technological literacy, and personality traits may provide deeper insights into the variability of user interactions with AI systems.

In summary, this study found that cognitive dissonance in the failure of AI services is deeply influenced by the way AI design explanations (intentional and design stances) and the nature of the tasks (emotional vs. mechanical) are presented. The theory of mental models underscores this relationship, illustrating how mismatched expectations manifest in user experience. In emotional tasks, explanations from an intentional stance lead users to expect interactions more similar to those with humans, thereby generating a stronger sense of dissonance when AI errs. In contrast, the design stance offers a clear, mechanical explanation for mechanical tasks’ outcomes, which, when unmet, indicates a gap in AI’s functional capabilities, thus affecting user trust and showing how a deviation from the expected framework drives cognitive imbalance. This study also demonstrates that users’ understanding of AI is a subjective mental model, and the presentation of AI is crucial [10]. The user experience of generative AI largely depends on the mental models constructed by users themselves. This study demonstrates the need for precise mental models of users in human–computer interaction, which is of significant importance for the design of artificial intelligence and the enhancement of user experience.

### 5.1. Theoretical Implication

This study explores the theory of mental models in human–computer interaction using cognitive neuroscience methods and examines the role of stance attribution in mental models with a view to understanding how mental models influence human–AI interaction. The study further explores the concepts of intentional stance and design stance in the psychology of artificial intelligence and analyzes the neural mechanisms of cognitive conflicts induced by mental models. In addition, this study designed a new experimental method based on the classical oddball paradigm and applied it to AI psychology research. By measuring brain neural activity at the millisecond level, the method circumvents the problem of recall bias that may exist in traditional self-report methods and provides a new perspective for studying human–computer interaction.

### 5.2. Managerial Implication

The findings of this study provide crucial guidance for the design of AI systems and their interactive interfaces. The design of different stance attribution explanations influences users’ mental models, thereby affecting their cognitive conflicts and emotional responses. Thus, designers of generative AI can incorporate appropriate stance attribution explanations in explanations of different task failures to mitigate users’ negative experiences. Adjustments based on stance fundamentally affect users’ mental models of AI products. Expectations arising from a user’s design stance (e.g., “This assistant can calculate the optimal response”) vis-à-vis the intentional stance (e.g., “This assistant can understand your needs”) differ. Through these indicators, companies can guide users toward a more tolerant and understanding attitude toward the limitations of artificial intelligence, enhancing user experience and satisfaction with human–computer interaction.

### 5.3. Limitations and Directions for Future Research

However, this study has its limitations, revealing, to an extent, the impact of generative AI stance attribution explanation on mental models through indirect evidence from cognitive responses across different task types, without a direct measure to better prove changes in the unconscious level of mental models. Additionally, the study categorizes service content into emotional and mechanical types, a broad classification method that may restrict the applicability of the research findings in real-world scenarios. Future research could pioneer new paradigms for measuring users’ unconscious mental models, such as exploring through brain–computer interface machine learning technologies. Further research could also divide AI service tasks into more detailed categories to investigate users’ expectations and experiences during different tasks. Additionally, future studies might consider the ethical implications and social impacts of stance attribution in artificial intelligence, such as understanding how different attributions affect dependence on or bias against AI systems. A key limitation of this study is the indirect nature of measuring unconscious cognitive processes. While EEG offers valuable insights into neural activity associated with cognitive conflict and error monitoring, it does not provide direct access to unconscious thought. The observed theta-band ERSP and PLV changes are interpreted as correlates of these processes, but further research using complementary methods, such as implicit association tests, could provide a more nuanced understanding of the unconscious mechanisms at play. Although the sample size of this study was validated by statistical efficacy analysis, we recognize that a larger sample size may lead to stronger robustness of results. Future studies should consider expanding the sample size to further validate the findings of this study. While this study focused specifically on Cz and F6 based on their established association with cognitive conflict processing, future research could explore the activity and connectivity of other electrodes to gain a more comprehensive understanding of the neural network dynamics underlying HCI. This broader perspective could reveal additional insights into the complex interplay of brain regions involved in AI stance attribution explanation. This study acknowledges certain limitations regarding the generalizability of its findings. We did not collect detailed demographic information or assess user experience with AI/LLM technology, which could influence their responses to system failures. Future research should explore the impact of user characteristics, such as prior experience with AI, tech savviness, and attitudes toward AI, on their reactions to LLM errors. As user familiarity and acceptance of AI/LLM technology evolve, their expectations and responses to system failures might also change. Longitudinal studies are needed to understand how user perceptions and behaviors adapt over time in response to advancements in AI technology.

## Data Availability

The original data presented in the study are openly available in OSF at https://osf.io/vbs43 (accessed on 15 October 2024).
